# Overexpressing *Ugp1* promotes phosphate uptake and accumulation in rice (*Oryza sativa*)

**DOI:** 10.1007/s12298-023-01368-8

**Published:** 2023-10-13

**Authors:** Wenqi Zhang, Qi Meng, Wei Liu, Pinzhu Qin, Bowen Li, Guohua Xu

**Affiliations:** 1 College of the Environment & Ecology, Jiangsu Open University, Nanjing, 210017 China; 2 State Key Laboratory of Crop Genetics and Germplasm Enhancement, Nanjing Agricultural University, Nanjing, 210095 China

**Keywords:** *Oryza sativa*, UDP-glucose pyrophosphorylase, Sucrose, Phosphorus, Phosphate starvation signaling

## Abstract

Plant responses to phosphate (Pi) starvation stress involve an array of adaptive strategies including enhanced accumulation and shoot-to-root transport of carbohydrates required for activating the plant Pi starvation signaling. However, the contribution of carbohydrate biosynthesis genes to maintaining phosphorus (P) homeostasis remains unknown, and the functional characterization of sugar metabolism genes is often impeded or compromised due to the loss of fertility of the null mutants. Here, a highly expressed gene encoding UDP-glucose pyrophosphorylase in rice (*Oryza sativa*), *Ugp1*, was functionally characterized. *Ugp1* was transcriptionally induced in leaf blades by Pi starvation. As a link between sucrose (Suc) and P, we assumed that overexpression of *Upg1* would alter the Suc concentration. Given that Suc is a signaling molecule, such concentration changes would affect the P deficiency signal transduction, thereby altering the homeostasis of endogenous P. The results showed that, overexpression of *Ugp1* decreased plant biomass, increased sucrose content, and promoted Pi accumulation. The elevated Pi accumulation in *Ugp1-OX* plants was accompanied by the up-regulation of Pi-starvation-induced genes. Taken together, these results demonstrate that *Ugp1* is a positive regulator of sucrose accumulation, and is required for maintaining P homeostasis in rice. *Ugp1* thus represents a novel entry point to dissect the mechanisms underlying the carbon-P crosstalk.

**Supplementary Information:**

The online version contains supplementary material available at 10.1007/s12298-023-01368-8.

## Introduction

Rice is an extremely important cereal crop, which is the staple food for more than half of the world’s population. Currently, about 160 million hectares of paddy fields all over the world produce about 500 million tons of rice annually. However, this amount is not enough to feed the ever-growing population. In order to meet the increasing global demand, sustained efforts have been made to improve rice yield (Agarwal et al. 2016; Lau 2017).

Phosphorus (P) is one of the major macro-nutrients indispensable for rice growth and development, and is involved in important physiological and biochemical processes, such as biosynthesis, energy transfer and signal transduction. The major form of P for plants to acquire from soil is inorganic orthophosphate (Pi). Because soluble Pi is easily converted to organic form by soil microorganisms or is fixed by metal cations such as calcium, magnesium, aluminum, and iron, its mobility and availability in the soil are poor. Phosphorus is one of the main limiting factors for plant growth in farmland and natural ecosystems (Chapin 1996; Raghothama 1999). To obtain higher yields and better quality of rice, chemical P fertilizers are usually applied each year at relatively high rates, which poses a potential environmental threat and exacerbates the shortage of nonrenewable P mineral resources worldwide (Gilbert et al. 1999). Therefore, a full understanding of the mechanism of Pi signaling and absorption in rice is crucial to improve P-use efficiency (PUE). Cultivating high PUE rice varieties that effectively utilize soil P pools (including residual fertilizer P) is an unavoidable choice in the context of development of sustainable agriculture and protection of environment.

The plant Pi starvation responses show extensive interactions with other pathways, such as carbon (C) metabolism. Many plants, including rice, accumulate carbohydrates in source tissues in response to low-phosphorus stress and transport them mainly in the form of sucrose (Hammond and White 2011; Meng et al. 2020; Yoon et al. 2021). It has been reported that sucrose play a regulatory role in P deficiency signal transduction in plants (McKinley et al. 2016). Sucrose participates in the physiological processes and metabolic pathways such as root elongation, root hair growth, acid phosphatase secretion, and auxin synthesis to adapt to low Pi availability via regulating gene expression as a systemic signaling molecule (Dasgupta et al. 2014; Li et al. 2017; Zou et al. 2018). The alteration of synthesis or transport of sucrose influences the response to P starvation in plants (Hammond and White 2011). Some studies showed that inhibition of sucrose unloading in phloem could affect the response of plants to low-P stress (Lloyd and Zakhleniuk 2004; Zhang et al. 2015). Our recent results indicate that knockout of *OsAGPL1* or *OsAGPS1*, two genes encoding the enzyme catalyzing the initial and rate-limiting step in starch biosynthesis, leads to a significant decrease in starch content and an increase in Pi accumulation. We think that it is due to a complementary increase in sucrose concentration because sucrose acts as a signal molecule influencing the P signaling pathway in rice, activates the expression of Pi-starvation-induced (PSI) genes, and finally modifies the uptake of Pi (Meng et al. 2020). However, the balance between starch and sucrose is very interesting. A change in the accumulation of either starch or sucrose may lead to an opposite or the same alteration in the other one (Miyagawa et al. 2001; Pal et al. 2013; Lee et al. 2014). To obtain robust evidence, we characterized the gene *Ugp1* through bioinformatics analysis and RNA-seq results in public databases (Secco et al. 2013). This gene encodes an enzyme that participates in sucrose biosynthesis, namely uridine diphosphate-glucose pyrophosphorylase (UGPase).

**Fig. 1:**
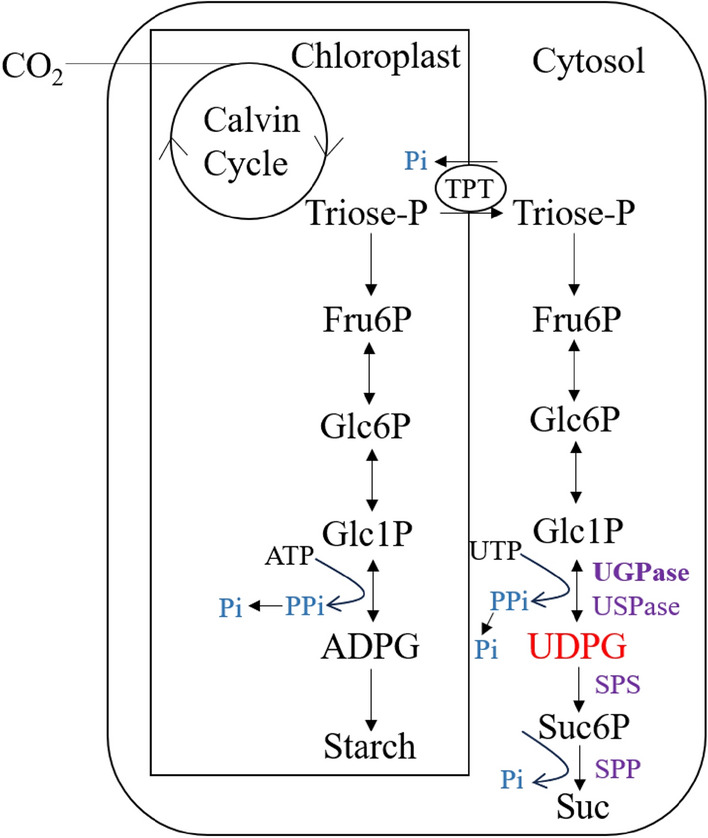
Main pathways of starch and sucrose synthesis in plant cells. Purple fonts indicate the enzymes involved in UDPG and sucrose formation. Arrows connect respective substrates to products. Abbreviations: Triose-P: Triose phosphate; Fru6P: Fructose-6-phosphate; Glc6P: Glucose-6-phosphate; Glc1P: Glucose-1-phosphate; ADPG: Adenosine diphosphate glucose; UDPG: Uridine diphosphate glucose; Suc: Sucrose; UGPase: UDPG pyrophosphorylase; USPase: UDP-sugar pyrophosphorylase; SPP: Sucrose phosphate phosphatase; SPS: Sucrose phosphate synthase

Sucrose synthesis in plants primarily occurs in the cytoplasm, using uridine diphosphate glucose (UDPG) as a precursor. Sucrose phosphate synthase (SPS) catalyzes UDPG and fructose-6-phosphate (Fru6P) to produce sucrose-6-phosphate, which is then hydrolyzed to form sucrose (Fig. 1). UDPG is crucial in various cellular process. Apart from serving as a precursor for sucrose, it is essential for cellulose, callose, and other cell wall polysaccharide synthesis (Kleczkowski et al. 2010; Bar-Peled and O’Neill 2011; Janse van Rensburg and Ende 2017). Additionally, UDPG acts as a glucose donor during the synthesis of glycoproteins, glycolipids, and sulfolipids (Okazaki et al. 2009). UDPG is known to participate in approximately 270 plant reactions (Chae et al. 2014). There are two enzymes, UGPase and uridine diphosphate-sugar pyrophosphorylase (USPase), that catalyze the reversible reaction using glucose-1-phosphate (Glc1P) as a substrate to synthesize UDPG (UTP + Glc1P ↔ UDPG + PPi) (Kleczkowski et al. 2010; Yin et al. 2011). In source tissues, UGPase primarily catalyzes UDPG synthesis. In mature *Arabidopsis* leaves (based on studies with UGPase mutants), UGPase accounts for at least 90–94% of UDPG-dependent activity (Park et al. 2010; Janse van Rensburg and Ende 2017). There are two types of plant UGPase, type-A and type-B. Type-A UGPase is typically cytoplasmic (Okazaki et al. 2009; Kleczkowski et al. 2010) and is particularly important in storage tissues (e.g., endosperm and tuber) and reproductive organs (Sowokinos et al. 1993; Spychalla et al. 1994). Some type-B UGPase enzymes localize to plastids and provide UDPG for sulfolipid biosynthesis (Okazaki et al. 2009). Consequently, UGPase plays a critical role in plant growth and development.

In rice, two UGPase homologs, *Ugp1* (LOC_Os09g38030) and *Ugp2*, encode type-A UGPase (Abe et al. 2002; Mu et al. 2009). *Ugp1* is abundantly expressed in pollen cells and maintains rice fertility by promoting callose deposition during the meiosis stage of pollen development (Chen et al. 2007b; Woo et al. 2008). *Ugp2* shows a much lower abundance compared with *Ugp1*, and is preferentially expressed in pollen (Chen et al. 2007b; Mu et al. 2009). Based on the bioinformatics analysis of *Ugp1* and the results of preliminary studies on the interaction between the carbon and P metabolic pathways (Chen et al. 2007a; Meng et al. 2020), it is hypothesized that *Ugp1* may participate in the P signaling pathway by (i) regulating the metabolic balance of endogenous carbohydrates, and (ii) influencing the crosstalk between the P and carbon metabolic pathways in rice. In this study, we overexpressed *Ugp1* in rice, and analyzed the growth, development, carbohydrate accumulation, and P acquisition of transgenic plants in different P supply conditions.

## Materials and methods

### Plant materials and growth conditions

A japonica variety (Nipponbare) of rice (*Oryza sativa* L. ssp. *japonica*) was used as the wild-type (WT). The transgenic plants and WT plants were grown in hydroponics providing standard rice culture nutrition as: 1.25 mM NH_4_NO_3_, 0.2 mM NaH_2_PO_4_, 0.513 mM K_2_SO_4_, 0.998 mM CaCl_2_, 1.643 mM MgSO_4_, 0.009 mM MnCl_2_, 0.075 mM (NH_4_)_6_Mo_7_O_24_, 0.019 mM H_3_BO_3_, 0.155 mM CuSO_4_, 0.02 mM Fe-EDTA, 1 mM NaSiO3, and 0.152 mM ZnSO_4_. Rice plants were grown in a growth chamber with 14 h light/12 h dark photoperiod, day/night temperatures of 30/22 °C, and the relative humidity was controlled at approximately 60%. Seedlings were treated with 0.5 strength of the nutrient solution described above until the fourth leaf blade just emerged. Subsequently, the seedlings were treated with the full-strength solution containing high phosphate (HP; 200 μM Pi; as detailed above) or low phosphate (LP; 10 μM Pi; 0.01 mM NaH_2_PO_4_) for 3 weeks during the vegetative stage, and then evaluated for phenotype or sampled.

### RNA extraction, cDNA synthesis and RT-qPCR

Total RNA was extracted from different plant tissues such as leaf blades, leaf sheaths and roots, using TRIzol reagent (Invitrogen). For synthesizing first-strand cDNAs from total RNA, we used a PrimeScript RT reagent kit with gDNA Eraser (TaKaRa Biotechnology, Dalian, China) according to the manufacturer’s instructions. Real time–quantitative PCR (RT-qPCR) was performed with a SYBR Premix Ex Taq™ II kit (TaKaRa Biotechnology) on a StepOnePlus Real-Time PCR System (Applied Biosystems) following the manufacturer’s instructions. Relative expression of the target genes was calculated against that of *OsActin1* (LOC_Os03g50885) with the 2^−△CT^ or 2^−△△CT^ method. All PCR primers used in this study are listed in Tables S1 and S2.

### Expression, purification, SDS/PAGE, and enzyme activity assay of UGPase

The *Ugp1* cDNA encoding sequence was subcloned into the pET-29a( +) vector (Novagen). The construct was transformed into *Escherichia coli* BL21/DE3 (pLysS) cells, and protein expression was optimized. The cells were grown to the late exponential phase (OD600 ~ 0.8) in conical flasks containing LB medium with 25 μg/mL kanamycin and 25 μg/mL chloromycetin. Recombinant protein expression was induced by the addition of isopropyl-d-thiogalactopyranoside (IPTG) to the final concentration of 1 mM. Cells were grown for another 8 h at 20 °C, and then centrifuged at 8000 g at 4 °C for 10 min. The pellet was resuspended in the binding buffer containing 20 mM Na_3_PO_4_, 500 mM NaCl and 5 mM imidazole. The homogenate was centrifuged at 10,000 g and 4 °C for 10 min, and the resulting supernatant was used to assay UGPase activity. The recombinant proteins were purified using a 6 × His-Tagged Protein Purification kit (CWBIO, CW0894S) based on manufacturer’s instructions. The protein concentrations were determined by a BCA Protein Assay kit (GenStar, E162-01).

The cell pellet or purified protein mixed with SDS loading buffer (Takara), was boiled for 10 min and then centrifuged at 10,000*g* for 5 min. The supernatant was loaded on a 12% SDS–polyacrylamide gel. After electrophoresis, the gel was stained with Coomassie blue. For Western blot analysis, proteins were transferred to a nitrocellulose membrane after electrophoresis. Membranes were blocked in 5% v/v non-fat milk in TBS-T (20 mM Tris–HCl, pH 7.6, 137 mM NaCl, and 0.1% w/w Tween 20) at room temperature for 1 h. For detection of recombinant protein, the membranes were incubated with an anti-6 × HIS antibody (CMCTAG, AT0025) at 1:5000 dilution with TBS-T at 4 °C overnight. Next, the membranes were washed in TBS-T three times for 10 min each. Membranes were then incubated for 1 h with a goat anti-mouse lgG (H + L) cross-adsorbed secondary antibody (Invitrogen, 35519) at 1:10,000 dilution in TBS-T, followed by five washes for 6 min each in TBS-T, and detection using DyLight 680.

The UGPase activity assay was performed with the recombinant proteins using a one-step spectrophotometric method (Sowokinos et al. 1993). The formation of NADPH was monitored at 37 °C until the maximum change in optical density occurred at 340 nm. The difference in absorbance before and after the addition of the supernatant was used to calculate the amount of NADPH generated. One unit of activity in the synthesis direction is defined as the amount of enzyme required to catalyze the production of 1 nmol of NADPH per minute.

### Vector construction for* Ugp1* overexpression, and generation of transgenic plants

For *Ugp1* overexpression, the GUSPlus reporter gene was introduced into pCAMBIA1305 via *Nco*I/*Bgl*II digestion, resulting in a new expression vector, pCAMBIA1305-GUSPlus. The *Ugp1* cDNA encoding sequence was amplified and fused upstream of the GUSPlus reporter gene via *Sac*I/*BamH*I. The constructs were introduced into *Agrobacterium tumefaciens* (strain EHA105) by electroporation. The *Agrobacterium*-mediated transformation was performed using mature embryos developed from seeds of WT rice plants following the standard procedure (Jia et al. 2011).

### Measurement of carbohydrates

Shoots and roots of WT and transgenic plants were sampled separately and dried. A sample of 0.1 g was extracted in 10 mL distilled water at 100 °C for 30 min. The extract was filtered into a 25 mL volumetric bottle, and the reaction mixture, containing 0.5 mL of extract, 1.5 mL of distilled water, 0.5 mL of 2% (w/v) anthrone with ethylacetate and 5 mL of 18 M H_2_SO_4_, was incubated in boiling water for 1 min. The washed precipitate, after digestion with amyloglucosidase, was used for starch estimation (Fredeen et al. 1989). Sucrose was estimated as described previously (Jelitto et al. 1992). All determinations were performed in four to six replicates from which standard deviation was calculated.

### Measurement of soluble Pi and total P concentration

Shoots and roots of WT and transgenic plants were sampled separately. For the measurement of soluble Pi concentration, about 0.5 g of fresh samples were used following the method below (Zhou et al. 2008). Soluble Pi was extracted by perchloric acid and determined by the molybdenum blue method. For the measurement of total P concentration, about 0.1 g of dry samples were used following the method described elsewhere (Chen et al. 2007a). The dried plant tissues were ground (average diameter 1 mm) and then digested with a mixture of 5 mL of 98% w/w H_2_SO_4_ and 3 mL of 30% v/v hydrogen peroxide. After cooling, the digested sample was diluted to 100 mL with distilled water. The total P concentration in the solution was measured using the molybdenum blue method with absorbance read at 820 nm on a SpectraMax M5 multidetection microplate reader system.

### Phosphorus uptake using ^32^P isotope

To perform the Pi uptake assay, 10-day-old WT and transgenic plants were treated with HP or LP nutrient solution for 7 days before ^32^P uptake assay. The plant roots were first incubated in a pretreatment solution (2 mM MES and 0.5 mM CaCl_2_, pH 6.0) before transferring them into a 1 L uptake solution (nutrient solution specified above plus 100 μM NaH_2_PO_4_, pH 6.0) containing [^32^P] orthophosphate (PerkinElmer) (8 µCi L^−1^) for 5 min, 30 min and 3 h. At the end of the uptake period, roots were washed three times with double-distilled H_2_O, and then were transferred into ice-cold desorption solution (2 mM MES, 0.5 mM CaCl_2_, 100 μM NaH_2_PO_4_, pH 6.0) for 15 min. Afterwards, the roots were blotted, plants were cut into shoots and roots with a razor, weighed, and placed in 10 mL centrifuge vials with 1 mL of perchloric acid and 0.5 mL of hydrogen peroxide. The vials containing samples were then digested in an oven at 65 ℃ overnight. Subsequently, 0.2 mL of the digest was transferred into a 5 mL vial, 3 mL of scintillation cocktail (ULTIMA GOLD™ LLT; PerkinElmer) was added to each vial, and radioactivity was measured by a scintillation counter (Beckman Coulter LS6500).

## Results

### *Ugp1* was specifically induced by phosphate deficiency and showed UGPase activity in vitro

**Fig. 2:**
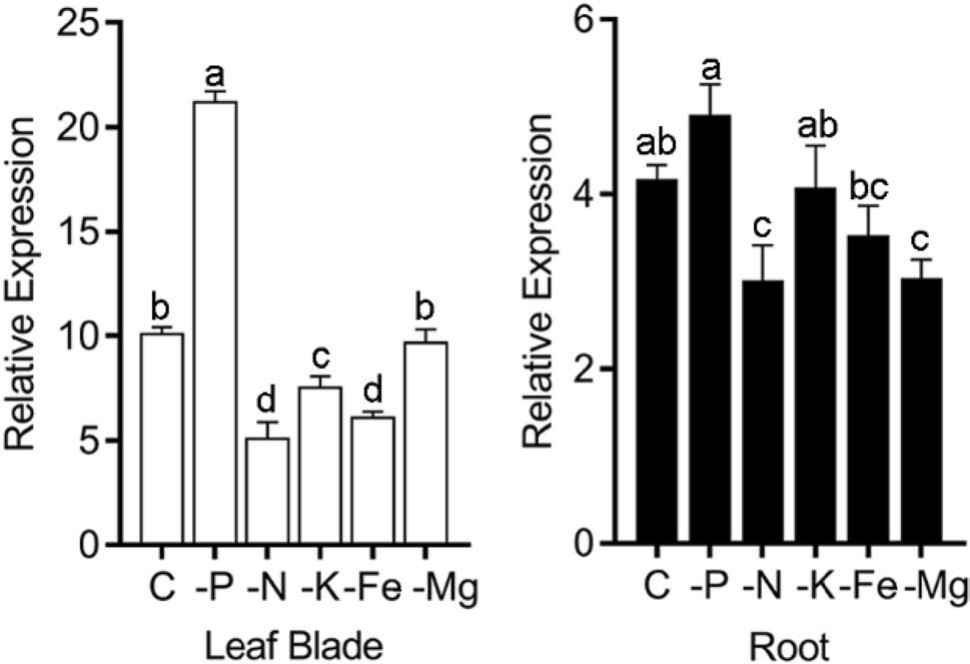
The Expression Pattern of *Ugp1*. The expression of *Ugp1* in response to deficiency of various nutrients. RT-qPCR was performed on total RNAs from the 5th leaf blade and root of 15-d-old seedlings after growth in nutrient sufficient (C), phosphate-deficient (-P), nitrate-deficient (-N), potassium-deficient (-K), iron-deficient (-Fe) and magnesium-deficient (-Mg) conditions for 10 d. Values represent means with SE of three biological replicates. Different letters indicate significant differences (*P* < 0.05, Duncan’s *t*-test)

We screened the reported transcriptomic data and found that *Ugp1* was up-regulated in rice shoots by Pi starvation stress. For validations, RT-qPCR analysis was performed to analyze the transcriptional response of *Ugp1* to major nutrient deficiencies. The results confirmed that *Ugp1* expression was induced by Pi starvation in leaves but not in roots. This transcriptional induction was not observed upon deprivation of nitrogen, potassium, magnesium or iron (Fig. 2).

**Fig. 3:**
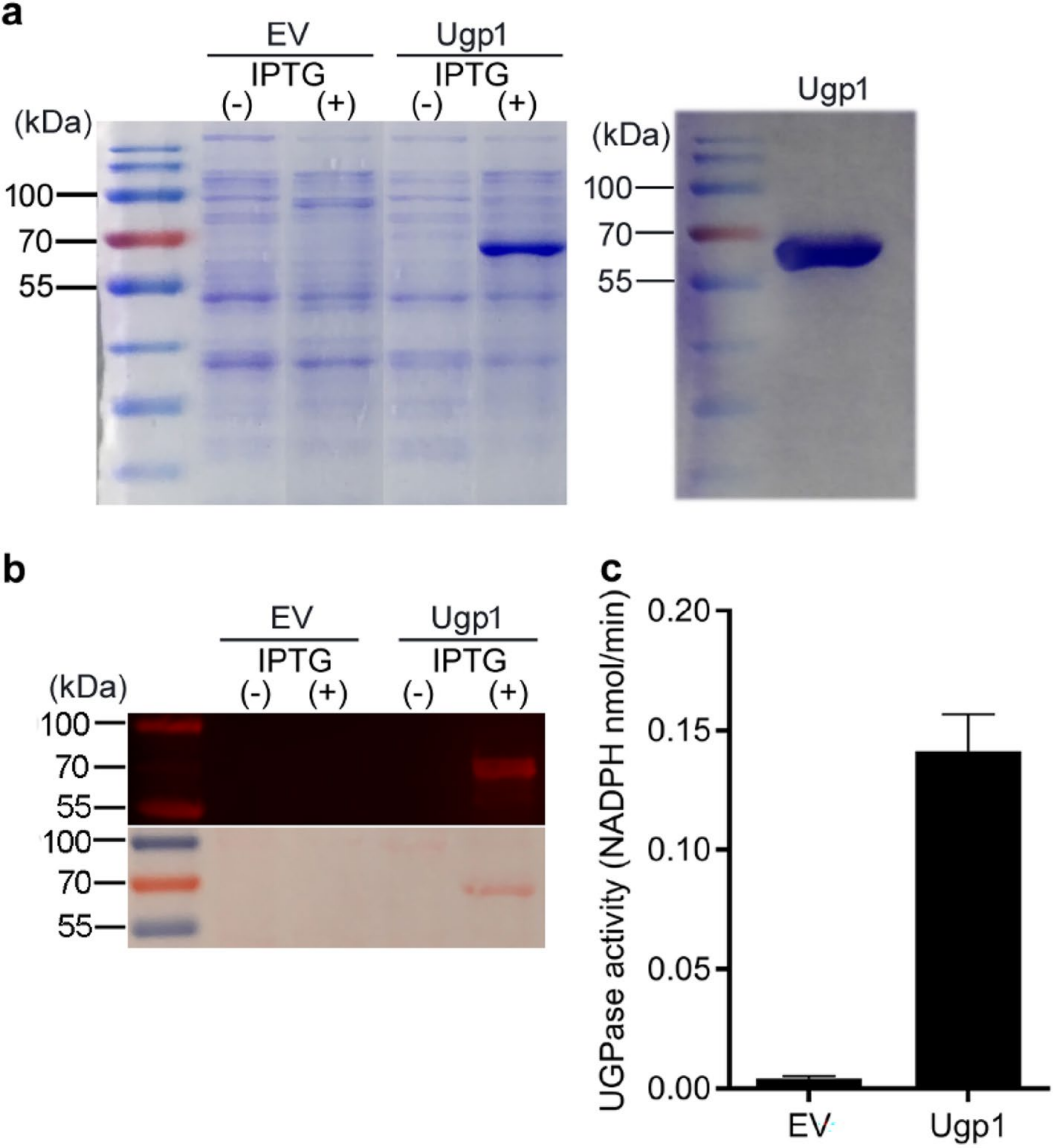
Expression, Purification and Enzyme Activity Assay of Ugp1 in *E. coli*. **a** SDS-PAGE of empty vector (EV) and recombinant proteins induced with ( +) or without ( −) IPTG (left) and purified recombinant Ugp1 protein (right). The molecular weight of the His-tagged vector (6.3 kDa) recombinant Ugp1 protein agrees with the estimated value of 51.6 kDa for the full-length Ugp1 protein (469 aa). **b** Western blot analysis of EV and purified recombinant proteins induced with ( +) or without ( −) IPTG. The proteins separated by SDS-PAGE were transferred to the PVDF membrane, and the membrane was incubated with His-tagged antibody and lgG (H + L) antibody successively. The specific bands could be observed at 680 nm (upper panel) or after Ponceau S staining (lower panel). **c** Activity assays of recombinant Ugp1 protein. The formation of NADPH was calculated from the absorption changes at 340 nm monitored after 4 h using an NADPH molar extinction coefficient of 6.22 × 10^3^ M^−1^ cm^−1^

The *Upg1* cDNA encoding sequence was firstly subcloned into the pET-29a vector and then transformed into *E. coli* for enzyme activity assays. The empty vector was used as a negative control. The expression of His-tagged recombinant protein was induced by adding IPTG, and the protein was purified and separated by SDS-PAGE (Fig. 3a). Western blot analysis was used to confirm whether the recombinant protein was expressed successfully in vitro (Fig. 3b). The UGPase activity assay was performed with recombinant protein by monitoring the NADPH formation at 340 nm. As expected, after the addition of IPTG, the Ugp1 recombinant protein was successfully expressed in *E. coli*. Furthermore, the enzyme activity of the recombinant protein was detected, whereas the empty vector protein had no enzymatic activity, indicating that the recombinant protein was functional (Fig. 3c). In recombinant protein, the UGPase enzyme activity was 0.14 nmol NADPH/min, which was approximately 28 times that of the empty vector protein.

### Identification of* Ugp1*-overexpressing transgenic rice plants

**Fig. 4:**
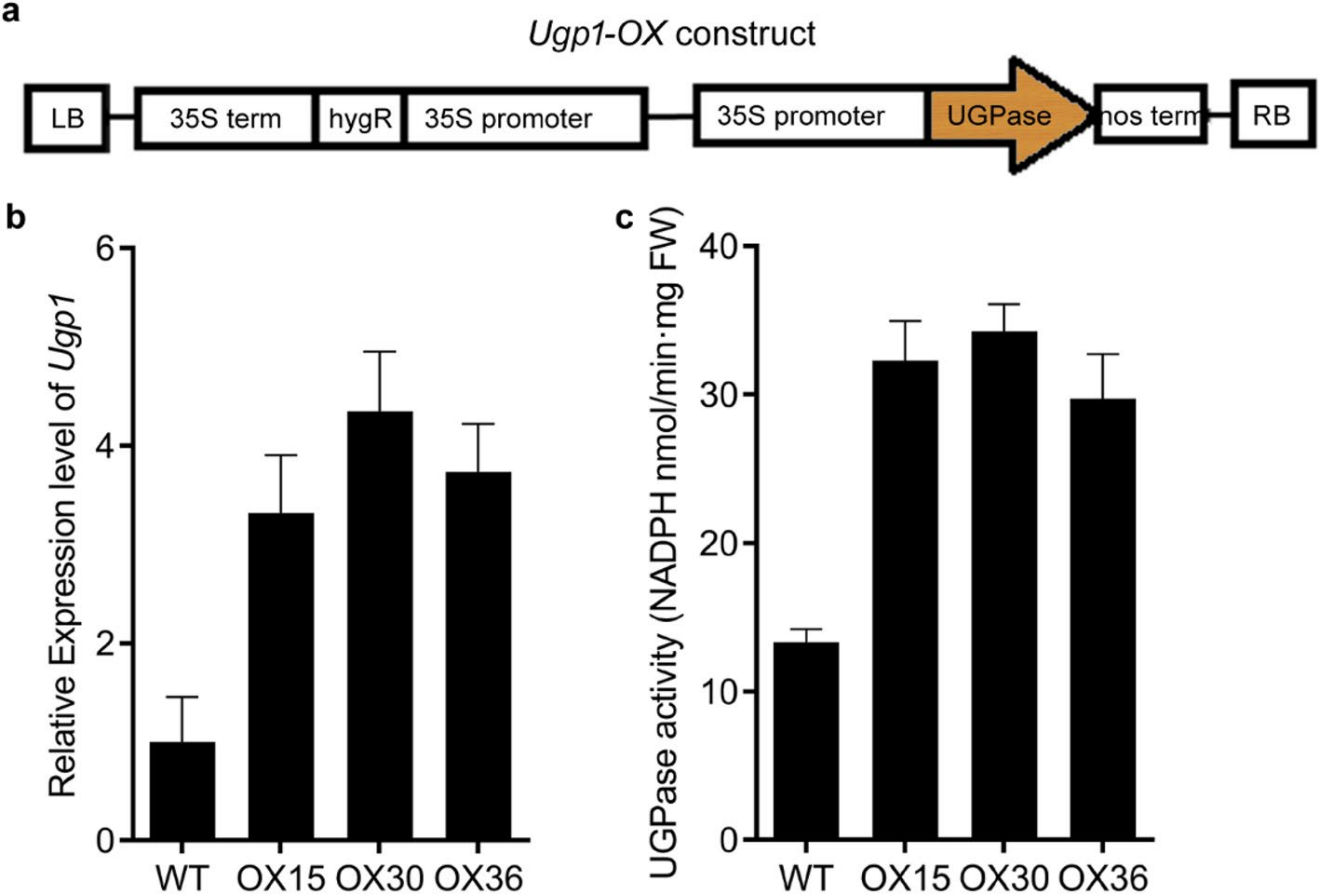
*Ugp1* Expression in transgenic rice plants. **a** Structure of *Ugp1*-overexpressing construct for rice transformation. The construct was developed under the control of the 35S promoter and the nopaline synthase (nos) terminator cassette. **b** Relative expression level of *Ugp1* in *Ugp1*-OX T1 generation transgenic plants. The data are means with SE of three biological replicates. **c** UGPase activity in transgenic plants. UGPase activities are shown as means with SE of three biological replicates

To elucidate the role of *Ugp1* in rice growth and development, we overexpressed the *Ugp1* gene under the control of the CaMV 35S promoter in the pCAMBIA1305.1 vector (Fig. 4a). About 40 independent transgenic plants (*Ugp1-OX)* were produced in T0 generation (Fig. S1). *Ugp1-OX* T0 plants with a high *Ugp1* expression level (about 10 lines) were selected for further characterization. T1 lines were germinated and transplanted in the field in Nanjing. Three lines selected randomly were used for seeding, named OX15, OX30 and OX36. When the seedlings grew to the four-leaf-stage in a controlled-environment chamber, samples of leaf blades were collected, and RNA was extracted to identify the abundance of *Ugp1* transcripts in transgenic plants (Fig. 4b). The results showed that the *Ugp1* expression significantly increased in these three lines. The expression levels of *Ugp1* in the OX15, OX30, and OX36 lines were 3.3, 4.3, and 3.7 times higher than that of WT rice, respectively. We also performed UGPase activity assays in transgenic plants (Fig. 4c). Compared with WT, *Ugp1-OX* T1 plants showed a 2.5-fold higher induction of UGPase activity in leaves.

### Overexpressing* Ugp1* affects rice growth and development

**Fig. 5:**
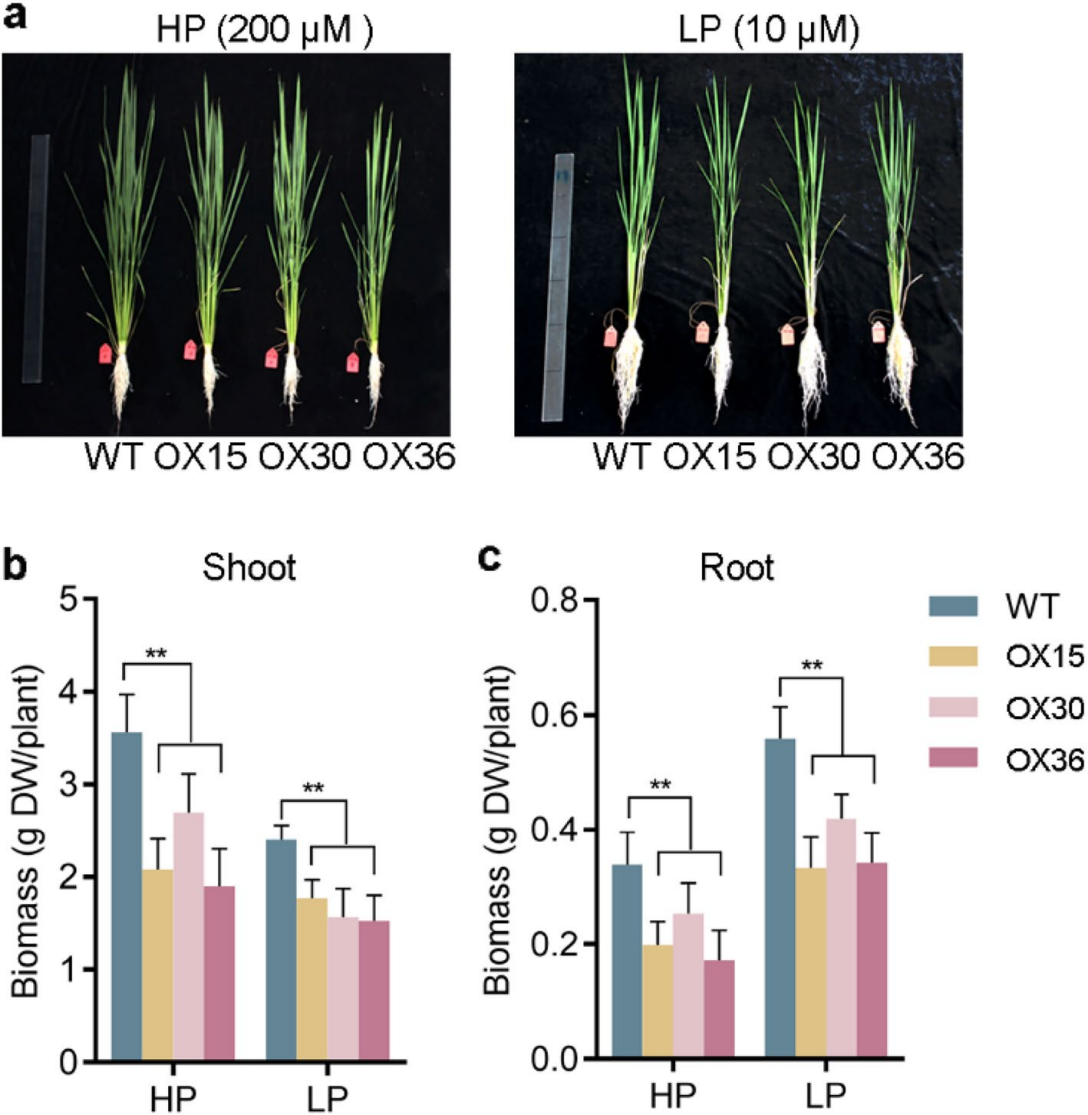
Phenotype of *Ugp1*-overexpressing plants. **a** Phenotype of *Ugp1*-overexpressing plants grown in nutrient solution supplied with different concentrations of Pi (HP, 200 µM Pi; and LP, 10 µM Pi) for 3 weeks. **b**, **c** Biomass of shoots and roots in *Ugp1-OX* lines under HP and LP supply. DW, dry weight. Values represent means with SE of four biological replicates, **P < 0.01, Duncan’s *t*-test

When *Ugp1-OX* transgenic plants were grown in soil until ripening, they displayed a phenotype of significantly shorter plant height than wild type (Fig. S2). However, this phenotype did not appear during the vegetative stage. To identify the influence of *Ugp1* on rice growth during vegetative stage, three independent *Ugp1-OX* lines (OX15, OX30 and OX36) were used to detect the biomass and length of shoots and roots in HP (200 μM Pi) and LP (10 μM Pi) conditions (Fig. 5a). Biomass of *Ugp1-OX* shoots was decreased in both HP and LP conditions in comparison with WT (Fig. 5b). Similarly, a decrease in root biomass was observed in the *Ugp1-OX* lines (Fig. 5c). In addition, it was observed that treatment with LP significantly promoted the growth of roots and had an inhibitory effect on shoots growth in both WT rice and *Ugp1-OX* lines, resulting in a significant increase in root-to-shoot ratio (Fig. 5b, c).

### Carbohydrate allocation is altered in* Ugp1*-overexpressing plants

**Fig. 6:**
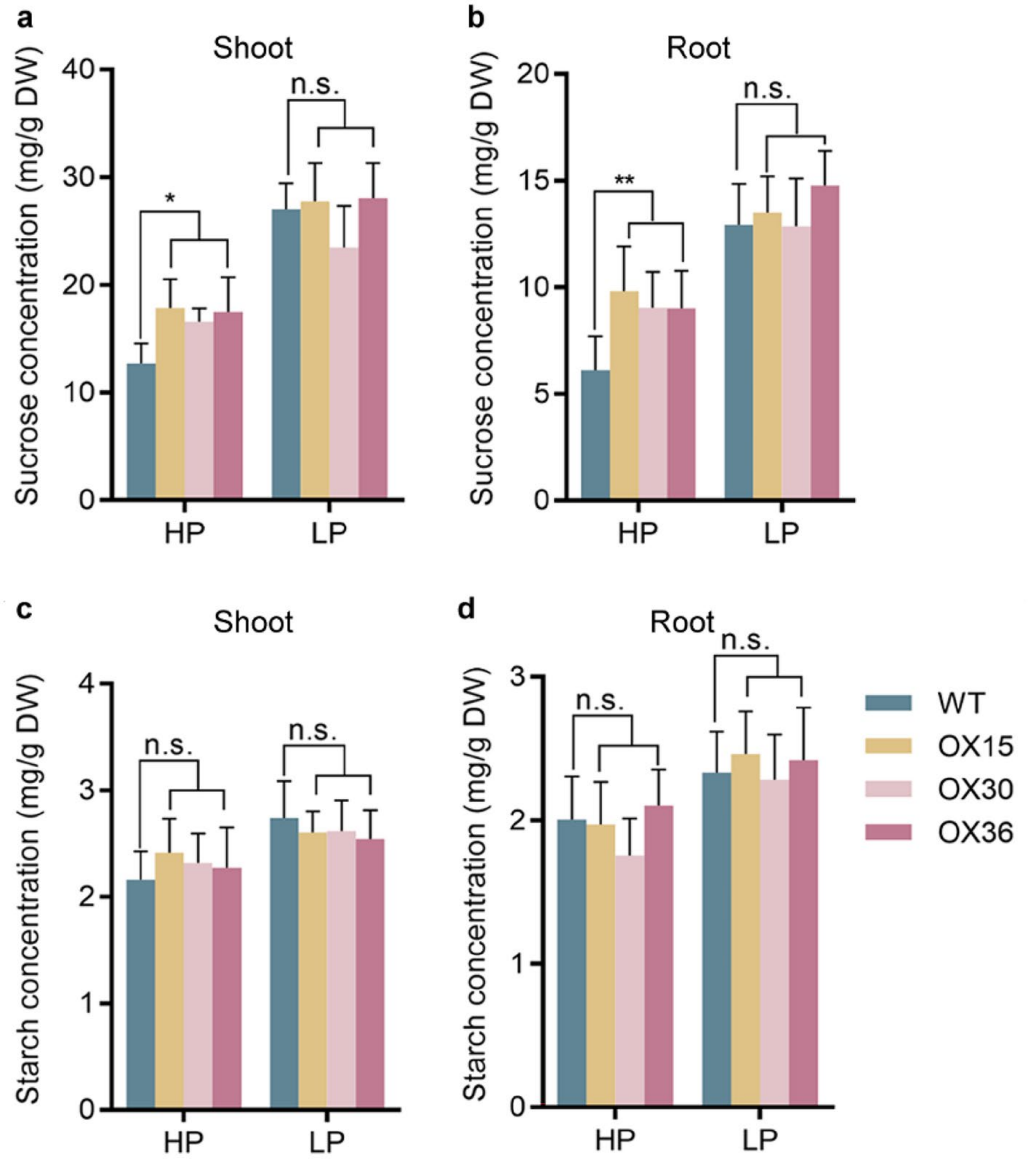
Carbohydrate Concentration in *Ugp1*-overexpressing plants. **a**, **b** Sucrose concentration of shoots and roots in *Ugp1-OX* transgenic plants under HP (200 μM) and LP (10 μM) supply. **c**, **d** Starch concentration in shoots and roots of *Ugp1-OX* transgenic plants under HP and LP supply. The seedlings were grown hydroponically for 3 weeks. DW, dry weight. Values represent means with SE of six biological replicates, *P < 0.05, **P < 0.01, Duncan’s *t*-test

The concentrations of sucrose and starch were determined in the roots and shoots of *Ugp1-OX* plants after 3-weeks of growth in HP and LP conditions to examine the effects of *Ugp1* overexpression on internal sugar levels (Fig. 6). Sucrose concentration was significantly higher in transgenic plants than WT plants grown with sufficient Pi supply. Interestingly, such an increase did not occur in the LP treatment. No change was observed in starch concentration regardless of HP or LP supply.

### *Ugp1* affects Pi uptake and accumulation in rice

**Fig. 7:**
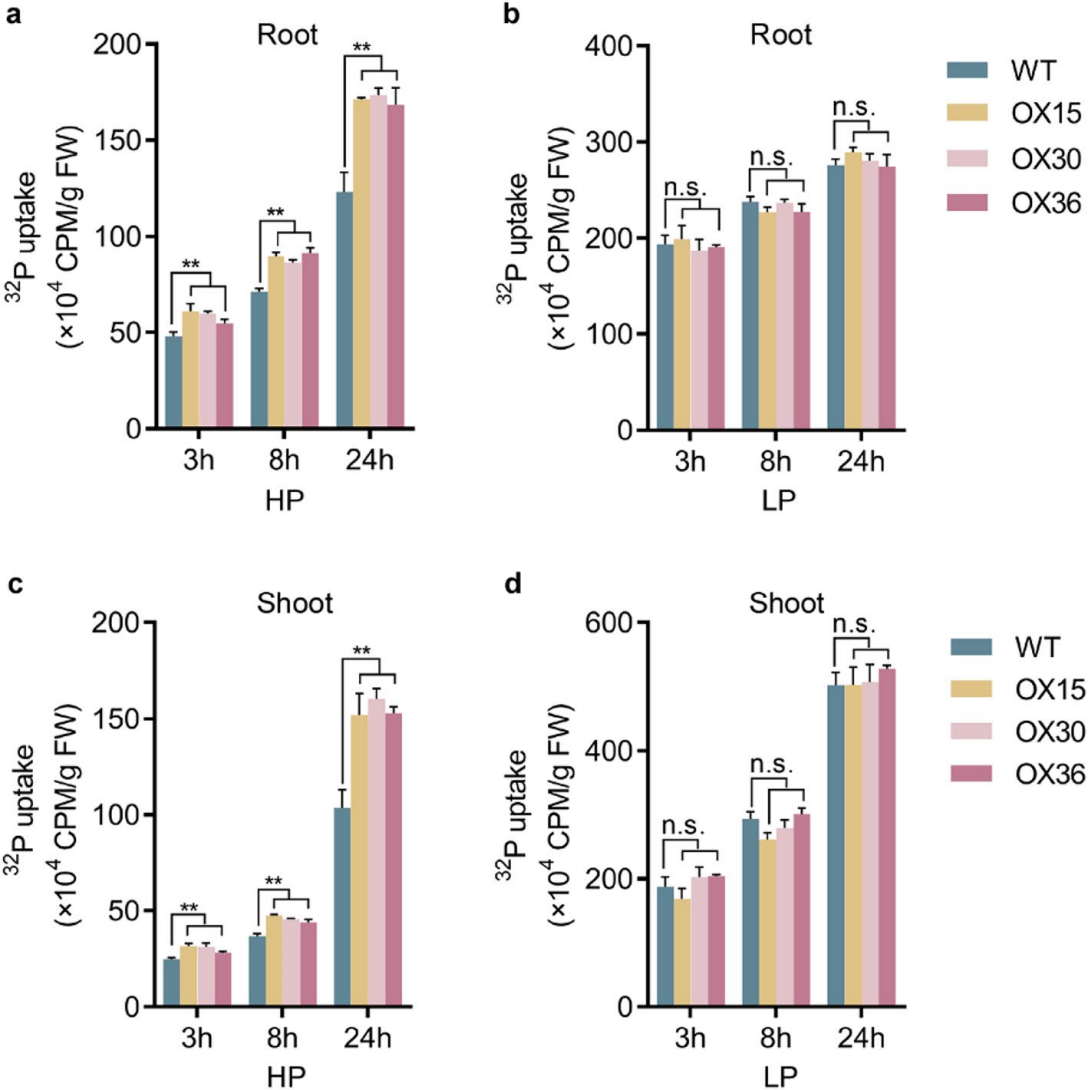
Pi Uptake rate in *Ugp1*-overexpressing plants. Pi uptake by roots and shoots of *Ugp1-OX* and WT plants. The seedlings were grown with Pi-sufficient (HP, 200 µM Pi) and Pi-deficient supply (LP, 10 µM Pi) for 3 weeks, and placed in a hydroponic system containing 100 µM Pi labeled with radioactive ^32^P. The Pi uptake were assessed after 3, 8 and 24 h. Values presented are means with SE of three biological replicates. FW, fresh weight. **P < 0.01, Duncan’s *t*-test

**Fig. 8:**
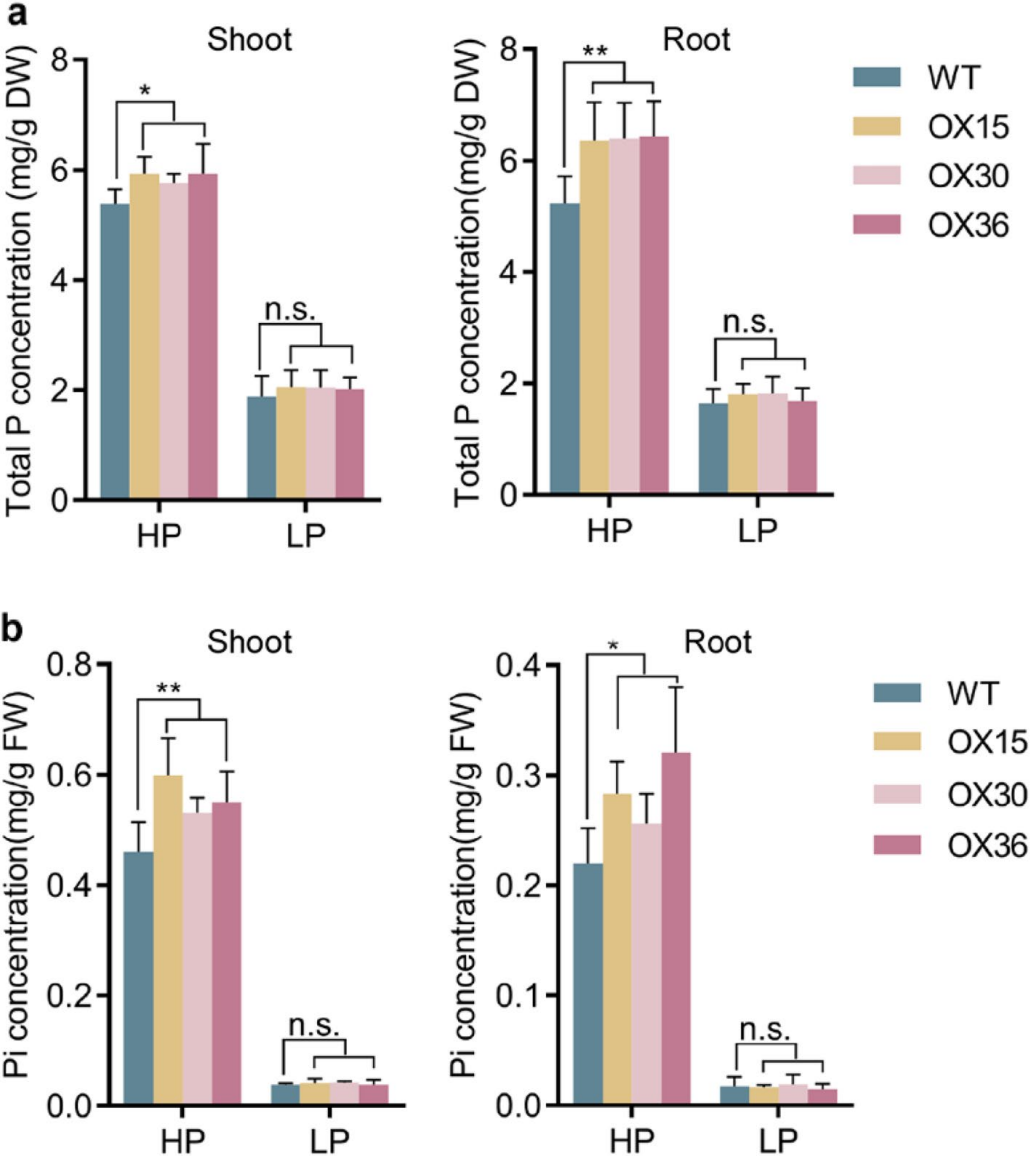
Total Phosphorus and Inorganic Phosphate Concentration in *Ugp1*-overexpressing plants. **a** Total P concentration of shoots and roots in *Ugp1-OX* lines and WT. **b** Pi concentration of shoots and roots in *Ugp1-OX* lines and WT. The plants were grown in the HP (200 µM Pi) and LP (10 µM Pi) nutrient solutions for 3 weeks. Values presented are means with SE of six biological replicates (A) and four biological replicates (B). DW, dry weight. FW, fresh weight. *P < 0.05, **P < 0.01, Duncan’s *t*-test

To investigate the potential role of *Ugp1* in maintaining Pi homeostasis, Pi concentration and ^32^P-labeled Pi uptake rate were detected in *Ugp1-OX* lines. Three-leaf transgenic and WT plants were grown in a hydroponic system with different Pi supplies for 3 weeks. Compared with WT plants, a significant increase in the short-term Pi uptake rate by *Ugp1-OX* roots as well as Pi accumulation in shoots was observed under HP but not LP supply, and the difference gradually increased over time (Fig. 7). After 24 h, Pi uptake rate of both roots and shoots in *Ugp1-OX* was more than1.4-fold higher compared to WT lines. Correspondingly, Pi concentration in shoots and roots was markedly higher in *Ugp1-OX* than WT plants when supplied with sufficient Pi (Fig. 8). Compared with WT plants, the concentrations of total P as well as inorganic Pi in transgenic lines were significantly (23 and 31%, respectively) higher in all tissues (especially in roots under HP supply). Under low P supply, the concentrations of total P and Pi did not differ between *Ugp1-OX* and WT plants. The results suggested a positive correlation between transcript levels of *Ugp1* and P concentration in rice treated with HP supply.

### Overexpression of *Ugp1* impacts the expression of *PHT1* genes

**Fig. 9:**
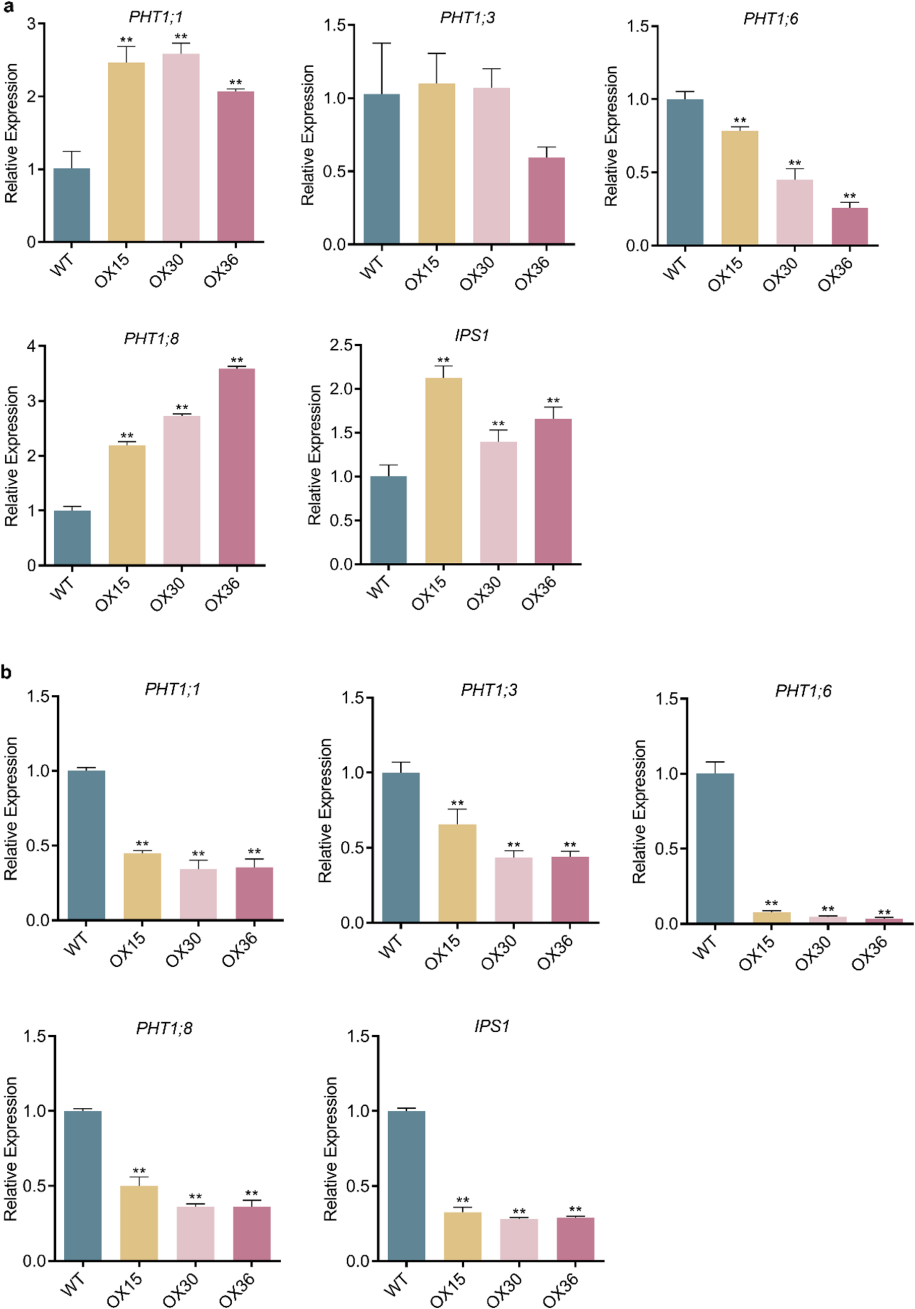
Expression of Genes Involved in Pi Transport and Signaling in *Ugp1*-overexpressing plants. The expression of major *PHT1* genes was measured in roots of *Ugp1-OX* transgenic lines and WT after treatment in the HP (200 µM Pi, a) and LP (10 µM Pi, b) nutrient solutions. Values presented are means with SE of three biological replicates. **P < 0.01, Duncan’s *t*-test

*Ugp1-OX* transgenic plants showed excellent Pi uptake capacity and thus accumulated more P than WT plants in the HP treatment. Did *PHT1* genes play a role in this process? To clarify the influence of *Ugp1* overexpression on *PHT1* genes, the expression patterns of several major *OsPHT1* genes were quantified (Fig. 9). As expected, the expression of all the detected genes (except *PHT1;6*) was induced in the *Ugp1-OX* lines when supplied with sufficient Pi (Fig. 9a). In particular, the expression of *PHT1;1* and *PHT1;8* (the two genes that play a crucial role in maintaining P homeostasis in rice) was up-regulated in transgenic plants in contrast to WT. Interestingly, although Pi uptake rate and Pi concentration in *Ugp1-OX* transgenic plants showed no significant difference compared to WT plants in LP conditions, there was a significant difference in the expression of *PHT1* genes. The *OsPHT1* genes were down-regulated (by at least 50%) in *Ugp1-OX* plants (Fig. 9b). These results suggested that *Ugp1* may participate in the response to Pi starvation by regulating the *PHT1* genes expression at the transcriptional level in normal P (HP) supply conditions.

## Discussion

### Ugp1 is a major UGPase for sucrose biosynthesis in rice

Sucrose is the main product of photosynthesis in plants. As a key enzyme in the sucrose synthesis pathway, UGPase has been studied thoroughly in various species from plants to microorganisms since 1953 (Munch et al. 1953; Sowokinos et al. 1993; Elling and Kula 1994). In the present study, we found that Ugp1 catalyzed the synthesis of UDPG in vitro, exhibiting UGPase activity (Fig. 3); the *Ugp1* overexpression resulted in increased sucrose accumulation in rice shoots and roots (Fig. 6). Similarly, overexpression of *LgUGPase* increased soluble sugar content in *Arabidopsis* (Mei et al. 2018). In addition, sucrose content in the *atugp1*/*atugp2* double mutant plants was decreased significantly compared to the wild type in *Arabidopsis* (Park et al. 2010). However, it is interesting to note that overexpression of the bacterial UGPase gene in hybrid poplar did not increase soluble sugar content (Coleman et al. 2007), which was likely due to the significant differences between the prokaryotic and eukaryotic UGPase genes, and the bacterial UGPase gene did not complement the function of poplar UGPase. In addition, the increase of sucrose concentration in leaf blades indirectly down-regulated the expression of a number of genes that encode the subunits and small subunits in the photosynthetic system, thus inhibiting photosynthesis (Hermans et al. 2006; Rook et al. 2006; Morcuende et al. 2007). This might explain the restricted growth of *Ugp1-OX* plants (Fig. 5). In summary, *Ugp1* plays an important role in carbohydrate metabolism involving sucrose biosynthesis; thus, overexpression of *Ugp1* can increase the endogenous sucrose content in rice.

### Intracellular sucrose is vital for maintaining P homeostasis in rice

In the past two decades, the mechanism of plant response to P deficiency signaling has been studied intensely, and sucrose plays an important role as a signaling molecule. The *Ugp1* is strongly and specifically induced in rice leaf blades by P deficiency but not by deficiency of other nutrients (nitrogen, potassium, iron, and magnesium) (Fig. 2). The *AtUgp1*, the homologous gene of *Ugp1* in *Arabidopsis*, can also be induced by low P. Given that the other product (in addition to UDP-Glc) of the reaction catalyzed by UGPase, namely PPi, could be further digested to Pi by pyrophosphatase (Segami et al. 2018), it could be postulated that the up-regulation of the *Ugp1* expression by P starvation can be considered as the biochemical regulatory mechanism for increasing Pi in plants.

P-starvation responses in plants involve increased sucrose content in source tissues. This accumulated sucrose in leaf blades can enhance the expression of SWEETs responsible for transporting sucrose to the roots. Finally, sucrose in roots can act as a systemic signal to regulate the expression of specific genes (Hermans et al. 2006; Nilsson et al. 2010; George et al. 2011). Once sucrose synthesis or transport is altered, the response of plants to P starvation can also change (Hammond and White 2011). When supplied with sufficient Pi, the ^32^P-labeled isotope absorption showed that Pi uptake as well as total P and Pi concentration was significantly higher in *Ugp1-OX* transgenic plants than in WT (Figs. 7, 8). The expression of major members of the *OsPHT1* family was detected through RT-qPCR, and the results showed that *OsPHT1;1* and *OsPHT1;8* were significantly up-regulated (Fig. 9a). These two genes are expressed almost constitutively in rice, and both participate in the uptake of Pi by roots and the transport and distribution of Pi in different tissues regardless of the level of P supply, playing a key role in maintaining P homeostasis in plants (Jia et al. 2011; Sun et al. 2012). In the presence of sufficient P supply, overexpression of *Ugp1* significantly promoted sucrose accumulation in rice shoots, increasing sucrose transport to and concentration in the roots. Given that carbohydrate concentration in root is crucial for responding to P starvation signals, the up-regulation of *PHT1* family genes followed, thereby promoting Pi uptake, transport and accumulation. This hypothesis is consistent with the results on *Arabidopsis*. The *athps1* mutants accumulated excessive sucrose in shoots and roots, making the mutant display many characteristics of the low-P response even when treated with sufficient P, including increased acid phosphatase activity, increased anthocyanin content, promoted lateral root development, and inhibited primary root elongation. In addition, about 73% of genes induced by P starvation in WT plants were up-regulated in the *athps1* mutant under sufficient P supply (Lei et al. 2011).

We found that the background expression level of *Ugp1* in WT plants was very high, with approximately 10 times the expression level of *OsActin* (OsRac1, AB047313) in leaf blades and more than 4 times in roots (Fig. 2). In addition, when subjected to P deficiency, the *Ugp1* expression was induced by more than twofold compared with the sufficient P supply. The expression level of *Ugp*1 was not completely linear with sucrose concentration. Studies have shown a threshold of UGPase activity for normal growth in the range of 6–25% of WT UGPase activity (Meng et al. 2009; Park et al. 2010). We also found a threshold for increasing UGPase activity, potentially explaining why overexpression of *Ugp1* did not alter the sucrose concentration under low-P stress (Fig. 6). When calculating the extent of increased carbohydrate concentration under P deficiency, we found that sucrose concentration in shoots of *Ugp1-OX* plants increased by 59% when treated with LP nutrient solution compared with the HP treatment, whereas this increase in WT rice was 100% (Fig. 6). This indicates that overexpression of *Ugp1* affects, at least partially, the response of rice to low P stress. The same result was also observed in roots as the sink tissue. The expression of tested *OsPHT1* family genes was down-regulated to varying degrees under LP supply (Fig. 9b). Considering the significant decrease in biomass of *Ugp1-OX* plants (Fig. 5), the unchanged Pi uptake rate and P concentration per unit weight might have been compensated for by biomass reduction (Figs. 7, 8). In summary, changing the expression of *Ugp1* affects the P homeostasis in rice, with *Ugp1* overexpression leading to P accumulation in rice under sufficient P supply.

It should be borne in mind that overexpression is not a usual approach for identifying gene function. This method was chosen due to the difficulty in obtaining *Ugp1* mutants. More convincing evidence is still needed to characterize *Ugp1* function. Moreover, due to the presence of *Ugp2*, the highly homologous gene to *Ugp1* in rice genome (Mu et al. 2009), further research remains to be done to determine whether *Ugp1* overexpression affects the expression and function of *Ugp2*, which in turn would influence the physiological and metabolic processes in rice.

### Supplementary Information

Below is the link to the electronic supplementary material.

Supplementary file1 (DOCX 1386 kb)
