# Supplementary material for: Overexpressing Ugp1 promotes phosphate uptake and accumulation in rice (Oryza sativa)
Source: Physiol Mol Biol Plants. 2023 Oct 13;29(10):1409–21. doi: 10.1007/s12298-023-01368-8 (PMC10709270; doi:10.1007/s12298-023-01368-8)
Supplement: Supplementary file 1 — Supplementary file1 (DOCX 1386 kb) [file 12298_2023_1368_MOESM1_ESM.docx]

**Supplementary Information**

**Table S1.** Primers Used in Constructs for Generating Transgenic Plants

| Primers | Sequence (5’ to 3’) |
| --- | --- |
| For overexpressing of *Ugp1* in rice | |
| Ugp1-Ox-F | ATGGCGGTCACCGCCGAC |
| Ugp1-Ox-R | TCAAAGATCCTCCGGAC |
| For identification of the *Ugp1-OX* plants | |
| Ugp1-Ox-q-F | GGAGCAGATCGAGTGGAGTAAGA |
| Ugp1-Ox-q-R | GAGCGTGTCGTAGGGAACCA |

**Table S2.** Primers Used for RT-qPCR Analysis

| Primers | Sequence (5’ to 3’) |
| --- | --- |
| Actin_qRT_F | GAGTCTGGCCCATCCATTGT |
| Actin_qRT_R | AGCATTCTTGGGTCCGAAGA |
| Ugp1-qRT_F | GGCTGCTCACGGAAACCTT |
| Ugp1-qRT_R | GCCGAATGCACACGACAAT |
| PHT1;1_qRT_F | CGCTTCCGTACGAGTGGTAGT |
| PHT1;1_qRT_R | GGTTCTTTCAAATCCAGGGAAA |
| PHT1;3_qRT_F | TGCGACTGCTGTATTCAGTACGT |
| PHT1;3_qRT_R | ACAAATGCCATCAAATATGAACAGA |
| PHT1;6_qRT_F | TATAACTGATCGATCGAGACCAGAG |
| PHT1;6_qRT_R | TGGATAGCCAGGCCAGTTATATATC |
| PHT1;8_qRT_F | AGAAGGCAAAAGAAATGTGTGTTAAAT |
| PHT1;8_qRT_R | AAAATGTATTCGTGCCAAATTGCT |
| IPS1_qRT_F | TTGGCAATTATTCGGTGGAT |
| IPS1_qRT_R | ACCATTTCACCATCCTCTTTATG |

Figure S1 Relative Expression Level of *Ugp1* in Transgenic Overexpression T0 Plants


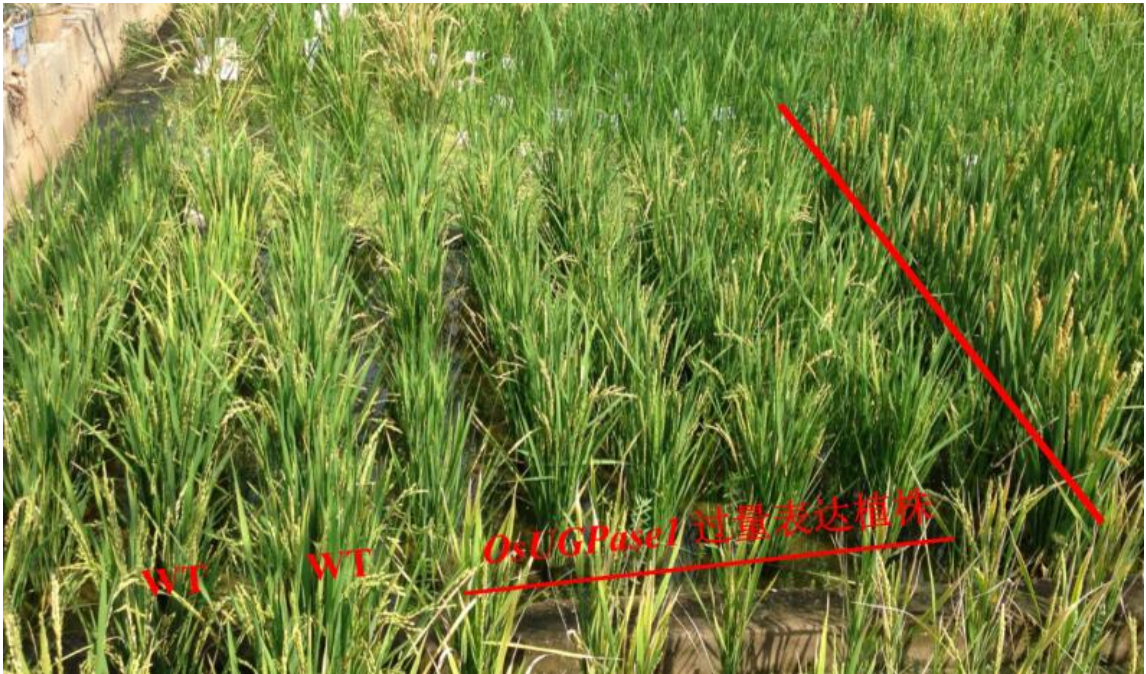


Figure S2 Phonotype of *Ugp1*-OX Plants in Field

The two rows on the left are wild type; the five columns on the right are *Ugp1*-OX plants.
